# Saccadic Movements in subjects with cerebellar disorders

**DOI:** 10.1590/S1808-86942010000100010

**Published:** 2015-10-17

**Authors:** Aloysio Augusto Tahan de Campos Netto, José Fernando Colafêmina

**Affiliations:** 1 Master's degree in otorhinolaryngology, Ribeirao Preto Medical School, Sao Paulo University (USP) / Ex-Fellow of The Ear Foundation at Baptist Hospital / The Otology Group - Nashville, Tennessee, EUA, Coordinator of the Otology and Cranial Base Surgery Group, Paulistana Santa Helena-Unimed Hospital / Doctoral student in Otorhinolaryngology, Ribeirao Preto Medical School, USP; 2 Associate Professor, Ribeirao Preto Medical School, USP. Associate professor of the Ophthalmology, Otorhinolaryngology and Head & Neck Surgery Department, Preto Medical School, USP

**Keywords:** cerebellum, cerebellar disorders, electronystagmography, saccades

## Abstract

Saccades are part of the electrooculography tests battery. The cerebellum has important connections with the brainstem and thalamic structures involved in the generation of saccades.

**Aim:**

to study saccadic movements in subjects with cerebellar disorders.

**Study Method:**

Prospective clinical study.

**Materials and Methods:**

11 subjects with cerebellar disorders were selected, together with a control group with 27 normal subjects. The patients of both groups had their saccadic movements registered (fixed and randomized). We compared and quantitatively analyzed the responses from both groups.

**Results:**

We did not find any differences among the quantitative parameters between the two. Age and gender did not influence these values. Despite these findings, the morphologies of the saccadic curves were very different between the two groups.

**Conclusion:**

Quantitative parameters of horizontal saccades from individuals with cerebellar diseases do not differ from those presented by normal subjects. Gender and age also did not influence these parameters.

## INTRODUCTION

The vestibular system is an extremely complex and fascinating object of study for neurophysiologists and otoneurologists worldwide. It includes not only the posterior labyrinth and vestibular nerves, but also central bodies such as the vestibular nuclei, the cerebellum, the thalamus, the frontal and prefrontal cerebral cortex, and other structures. The vestibular system coordinates balance, movement and gait. Information reaching the vestibular complex originates from the vestibular, visual, somatosensory and cerebellar systems. Balance is a complex physiological function requiring coordination and interaction among vestibular, visual, and somatosensory information, central compensation after injury, and anatomical and functional integrity of the central nervous system.^1^

Ocular motor control is useful for assessing the vestibular system because of the neural pathways that connect vision and the vestibular system. Ocular motor testing measures the accuracy, latency and velocity of eye movements to a given stimulus.

Computerized electronystagmography (ENG) is a diagnostic test for studying labyrinth disorders - after a detailed clinical history and physical examination - to help locate the injury site (peripheral, central or mixed) causing symptoms.

The oculomotor test battery consist of: saccadic tests, tracking, optokinetic nystagmus, and ocular fixation suppression tests.^2^

Saccadic movements are the main target of this study, and are one of the tests in ENG.

Saccades are rapid ocular movements that help redirect our line of vision; they consist of voluntary and involuntary fixation changes, the rapid phase of optokinetic nystagmus, the rapid eye movements (REM) of sleep, and the fast component of post-caloric nystagmus.

Saccades (or saccadics) are among the best-studied ocular movements; their dynamic properties are easily measured.

Saccades offer the possibility of investigating motor control, cognition and memory, and are commonly used with other techniques, such as functional image diagnostic methods and transcranial magnetic stimulation.^3^

Voluntary saccades in primates are associated directly with the presence of a fovea, in which images are better visualized. It is therefore unnecessary to move the head to visualize images located on the fovea. There are three relevant parameters for evaluating saccadic movements: peak velocity, latency and accuracy.

Peak velocity is the maximum velocity that eyes attain during saccades. Latency anomalies are the time difference between presentation of a target (light) and the onset of a saccadic movement; these anomalies comprise prolonged latency, shortened latency, and significant differences between right and left eye latencies. These anomalies are seen in neurodegenerative disease.^2^

Saccadic accuracy is determined by comparing the position of a patient's eye relative to the position of a target (light source). An eye saccadic movement moving beyond a point of light is named a hypermetric saccade or an overshoot dysmetria. A smaller eye saccadic movement than the position of the target of light is named a hypometric saccade or undershoot dysmetria. Saccadic tests may at times reveal cerebellar and central nervous system degenerative disorders, although the clinical examination is more effective for this diagnosis; saccades are an extra tool that otoneurologists have to make a correct diagnosis.

The most common cerebellar injuries are: toxins (ethanol, chemotherapy, anticonvulsants), auto-antibodies (paraneoplastic cerebellar degeneration, autoimmune diseases), structural lesions (ischemia, multiple sclerosis, tumors), and congenital and acquired cerebellar diseases. Acquired cerebellar diseases may be genetically determined. Subjects with the following acquired cerebellar degenerative diseases were investigated in this study: familial episodic cerebellar ataxia and Friedreich's ataxia.

The cerebellum gauges the amplitude of saccades (dorsal vermis and fastigial nucleus), apparently controlling the size of the saccadic pulse. The flocculus, and possibly the paraflocculus, appears to combine saccadic stimuli and pulses.^4^

The cerebellum appears to control saccadic accuracy and to correct position-dependent changes in the mechanical properties of the ocular muscles and orbitary tissues.

Studies on the relation between the cerebellum and saccadic movements are presented next.

Aschoff and Cohen (1971) showed that spontaneous horizontal saccades in monkeys were altered following unilateral injury of the cerebellar cortex.^5^

Optican and Robinson (1980) showed that although monkeys retained the ability to perform saccades in all directions and amplitudes after partial and total cerebellectomies, their saccadic pulse was no longer appropriate to the movement of the target.^6^

Vilis and Hore (1981) implanted probes in the fastigial nucleus (medial probe) and the dentate nucleus (lateral probe) of the cerebellum of Cebus monkeys, and produced probe-induced reversible lesions. Stimulation of lateral probes did not change the accuracy of horizontal and vertical saccades, but heat transmitted through the medial probe caused dysmetria, the magnitude of which depended of the eye position and the direction of saccades.^7^

Fuchs, Farrel and Straube (1993) studied monkeys and suggested that the function of the caudal fastigial nucleus of the cerebellum is to help accelerate contralateral saccades and to decelerate ipsilateral saccades.^8^

Barash et al. (1999) studied the effects of minor lesions of the oculomotor vermis of the cerebellar cortex on the ability of monkeys to execute and adapt eye saccadic movements. Their findings were that for horizontal saccades such lesions resulted in initial gross hypometria and a permanent abolition of the ability for rapid adaptation.^9^

Guan, Eggert, Bayer and Buttner (2004) studied the characteristics of saccades for static and migrating targets in three monkeys (Macaca mulatta) and concluded that the superior colliculus controls saccadic acceleration in response to mobile targets, and the cerebellum fine-controls deceleration changes.^10^

These studies have shown that the cerebellum considerably influences the neural pathways of saccadic movements. The controversies and complexities of cortical, brainstem and cerebellar connections involved in generating saccades have led us to believe that little is known about these ocular movements, and that further experimental work and models - including neural research - is required to deepen the scientific knowledge about this interesting field in otoneurology and neurophysiology.

The purposes of this study were as follows: to assess the features of saccadic movements by using ENG in patients with cerebellar conditions; to compare the three evaluation saccadic movement parameters (peak velocity, latency and accuracy) among subjects with cerebellar dysfunction and normal controls; and to record any differences in these parameters relative to sex and age in subjects with cerebellar conditions and normal controls.

## MATERIAL AND METHOD

Eleven patients with cerebellar dysfunctions seen at the neurology outpatient unit of a major university hospital were selected. Patients were referred to the otoneurology outpatient unit of this hospital to undergo ENG with an emphasis on saccades (the main object of this study).

There were four female and seven male patients. Ages ranged from 16 to 53 years; the mean age was 36.18 years.

Cerebellar diseases in this group were the following: familial periodic cerebellar ataxia (6 subjects of a same family and two of another), acquired cerebellar ataxia or Friedreich's ataxia, (two subjects), and one subjects with a congenital cerebellar condition of unknown etiology.

A control group consisting of 27 subjects with no cerebellar or labyrinthic complaints was also selected from accompanying family members or friends of patients visiting the otorhinolaryngology unit, undergraduate and graduate medical students. There were 15 female and 12 male subjects aged from 16 to 50 years (mean age 27.26 tears).

All subjects signed a free informed consent form (two copies), one in possession of the subject and the other of the researcher.

The hospital Institutional Review Board analyzed and accepted the project. Protocol number: 7770/2004.

ENG (and saccades) was carried out in a dark and silent room in the otoneurology laboratory of the otorhinolaryngology unit.

The ENG unit belongs to the otoneurology laboratory of the university hospital. The recording device was a 4-channel electrodiagnostic system (Micromedical Technologies Inc. US). The software was the Visual Eyes 4 for Windows 98.

Horizontal saccades were evaluated in all subjects, starting with the 10° fixed movements followed by 15° randomized movements. Vertical saccades were not assessed in this study.

**Figure 1:**
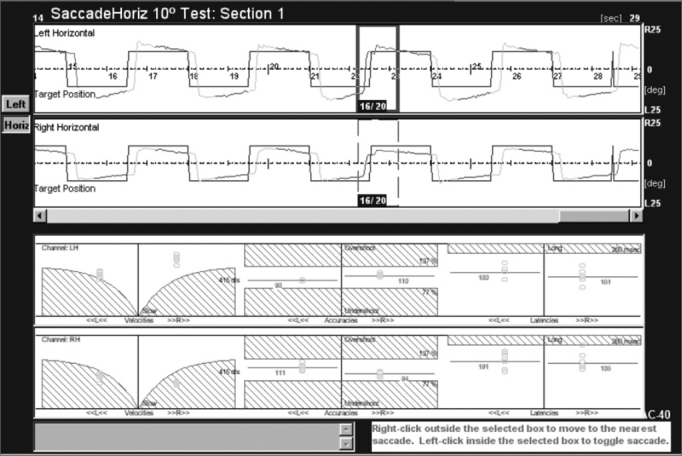
Tracings of saccades in the right and left eye and velocity, latency and accuracy charts on the lower portion of the figure. Control group patient.

The velocity chart shows the peak velocity on the Y axis (from 0 to 600°/s) and up to 50° deflection of the light point on the X axis for each saccade. Abnormally low velocities appear in dashed lines, which are a standard deviation below the mean. A mean velocity line is traced from velocity circles in each direction. This line helps analyze the mean velocity for each patient.

An accuracy chart shows the normal range (77 to 137%), where 100% is exact accuracy, more than 100% means that eyes stopped after the position of the light source, and less than 100% means that eyes stopped moving after reaching the position of the light source.

Latency was defined as the time from presentationof the target light source on the test bar and the beginning of the saccade to find it. All patients have a specific latency, measured in milliseconds.

Peak velocity, latency and accuracy values in both groups were obtained from the arithmetic mean of the most representative values in each response cycle of accepted responses by the ENG unit (Micromedical) for the control and study groups.

Findings in normal subjects (control) and patients with cerebellar conditions were analyzed within and among the groups. The main saccade analysis parameters (peak velocity, latency and accuracy) were analyzed statistically and presented in charts; the stationary and moving saccade tests were compared between the control and study groups. Age and sex were also analyzed relative to the three saccade parameters in both groups. These data were compared with other published results in the international literature.

The statistical method in this study for quantitative data (velocity, latency and accuracy) was the multiple linear regression model; according to statisticians, this model is more appropriate for comparing study and control groups for studying stationary and moving saccades, adding sex and age variables and analyzing the three parameters in two groups.

The multiple linear regression model was used for evaluating the differences between the study group (with cerebellar dysfunction) and the control group; this method is an extension of the simple linear regression model, and makes it possible to study the relation between several different variables (in this case: group, age and sex) and the response variable.^11^

Data is first explored to observe their behavior, after which the results of the multiple linear regression model are presented.

The significance level was 5%.

## RESULTS

The following results were found in the study group (with cerebellar conditions): for fixed saccades the mean velocity was 317.70°/s (SD - 74.40), ranging from 234.70 to 462.30; the mean latency was 188.60 ms (SD - 43.20), ranging from 137.40 to 307.50 ms; the mean accuracy was 101.61% (SD - 10.41), ranging from 90.10 to 127.70%.

Randomized saccades in the study group were as follows: the mean velocity was 326.60°/s (SD - 91.90), ranging from 243.50 to 498.70°/s; the mean latency was 192.50 ms (SD - 51.20), ranging from 144.70 to 325.20 ms; the mean accuracy was 98.63% (SD - 6.94), ranging from 88.60 to 113.90%.

Thus, the mean values of the three parameters are within normal limits according to the instruction manual of the device (Micromedical), as follows: peak velocity. 260°/s, accuracy from 77 to 137%, and latency. 100 ms. The accuracy/latency ratio is from 0.5 to 1.3.

The following fixed saccade values were found in the control group: mean velocity - 320.60°/s, SD - 54.90 ranging from 216.30 to 440.40°/s; mean latency - 158.89 ms, SD - 23.00 ranging from 98 to 199.00 ms; mean accuracy - 104.80%, SD - 3.98 ranging from 95.90 to 111.70%.

The following randomized saccade values were found in the control group: mean velocity - 304.50°/s, SD - 71.10 ranging from 144.60 to 436.40°/s; mean latency - 184.91 ms, SD - 21.02 ranging from 147.40 to 230.40 ms; mean accuracy - 101.25%, SD - 4.25 ranging from 91.00 to 109.40%.

In this group, the mean values of the three parameters are within normal limits according to the instruction manual of the device (Micromedical).

The mean velocity and median values of fixed saccades were slightly higher in the control group (320.60 and 317.60°/s) compared to the study group (317.70 and 301.80°/s) (Table 1).

**Table 1:** Description of the variables velocity, latency and accuracy for each group.

Variable	N	Mean	SD(a)	VC(b)	Minimum	Median	Maximum
**Cerebellopaths**							
*Fixed*							
velocity	11	317.70	74.40	23.40	234.70	301.80	462.30
latency	11	188.60	43.20	22.92	137.40	185.80	307.50
accuracy	11	101.61	10.41	10.24	90.10	100.90	127.70
*Randomized*							
velocity	11	326.60	91.90	28.13	243.50	293.60	498.70
latency	11	192.50	51.20	26.58	144.70	169.80	325.20
accuracy	11	98.63	6.94	7.03	88.60	97.80	113.90
**Normal**							
*Fixed*							
velocity	27	320.60	54.90	17.12	216.30	317.60	440.40
latency	27	158.89	23.00	14.48	98.00	161.30	199.00
accuracy	27	104.08	3.98	3.82	95.90	103.60	111.70
*Randomized*							
velocity	27	304.50	71.10	23.34	144.60	313.10	436.40
latency	27	184.91	21.02	11.37	147.40	183.10	230.40
accuracy	27	101.25	4.25	4.20	91.00	100.30	109.40

(a) SD: standard deviation;

(b) VC: variation coefficient (VC = SD/Mean).

Mean velocity, latency and accuracy values of the stationary and moving tests were higher in males compared to females in the study group.

Mean velocity values of the stationary and moving tests were slightly higher in females compared to males in the control group.

Mean latency values of the stationary and moving tests were slightly higher in males compared to females in the control group.

Mean accuracy values the stationary and moving tests were practically identical in both sexes in the control group (Table 2).

**Table 2:** Description of the variables velocity, latency and accuracy per group and according to sex.

Variable	n	Sexo	Mean	SD	VC	Minimum	Median	Maximum
**Cerebellopaths**								
*Fixed*								
velocity	7	male	341,80	81,70	23,91	253,20	317,70	462,30
	4	female	275,70	36,90	13,38	234,70	273,00	321,90
latency	7	male	198,30	50,40	25,42	159,20	187,50	307,50
	4	female	171,60	23,20	13,55	137,40	181,20	186,60
accuracy	7	male	103,66	12,72	12,27	90,10	102,40	127,70
	4	female	98,03	3,30	3,36	95,20	97,40	102,10
*Randomized*								
velocity	7	male	358,50	102,80	28,67	243,50	314,40	498,70
4	female	270,80	22,00	8,12	245,40	272,10	293,60	
latency	7	male	206,80	59,90	28,95	144,70	192,30	325,20
	4	female	167,40	15,38	9,19	146,90	169,25	184,20
accuracy	7	male	101,17	7,17	7,09	91,40	98,90	113,90
	4	female	94,18	4,00	4,24	88,60	95,15	97,80
**Normal**								
*Fixed*								
velocity	12	male	324,40	65,80	20,27	216,30	333,80	436,90
	15	female	317,50	46,60	14,68	230,20	316,90	440,40
latency	12	male	163,55	20,80	12,71	128,90	164,50	198,60
	15	female	155,15	24,68	15,91	98,00	158,00	199,00
accuracy	12	male	103,23	4,22	4,09	95,90	103,30	108,80
	15	female	104,77	3,78	3,60	100,00	105,40	111,70
*Randomized*								
	12	male	290,30	72,60	25,00	144,60	292,80	399,00
velocity	15	female	316,00	70,20	22,22	203,10	325,20	436,40
latency	12	male	188,06	16,86	8,96	158,00	186,50	212,20
	15	female	182,4	24,13	13,23	147,4	175,7	230,4
accuracy	12	male	101,32	4,75	4,69	91,00	100,80	109,00
	15	female	101,19	3,98	3,93	96,00	99,80	109,40

The multiple linear regression model for stationary and moving saccades was applied to obtain the following comparisons: study vs. control, and female vs. male for the three variables (velocity, latency and accuracy).

There were no differences between males (p-value 0.5694) and females (p-value 0.7709) when comparing subjects in the study and control groups for the variable velocity in the fixed test. There were no differences between females and males when comparing the study (p-value 0.7613) and the control (p-value 0.3273) groups.

There were no differences between females (p-value 0.4640) and males (p-value 0.8653) between the study vs. control groups for the variable latency (fixed test). Comparing females vs. males, there were no differences between subjects in the study (p-value 0.6089) and control (p-value 0.6369) groups.

There were no differences between females (p-value 0.8193) and males (p-value 0.2009) for the variable accuracy in the fixed test when comparing the study vs. control groups. Comparing females and males, there were also no significant differences between subjects in the study (p-value 0.9967) and control (p-value 0.5921) groups.

There were no significant differences between female (p-value 0.5178) and male (p-value 0.6436) subjects for the variable velocity in the randomized test when comparing the study and control groups. Comparing females and males also did not reveal differences between subjects of the study (p-value 0.8844) and control (p-value 0.3432).

There were no differences between females (p-value 0.5878) and males (p-value 0.5348) for the variable latency (randomized test) when comparing the study and control groups. Comparing females vs. males did not show differences between subjects in the study (p-value 0.7074) and control (p-value 0.9356) groups.

Again, there were not differences between females (p-value 0.4699) and males (p-value 0.6557) for the variable accuracy (randomized test) when comparing the study and control groups. Comparing females and males did not reveal differences between the study (p-value 0.4787) and control (p-value 0.6446) groups.

The statistical analysis revealed no quantitative differences in the parameters velocity, latency and accuracy among subjects with cerebellar diseases and normal subjects. However, there were qualitative differences between both groups in the morphology of tracings of stationary and moving saccade tests; subjects with cerebellar disease presented saccade dysmetria, correction saccades, glissades and other changes not found in normal subjects. These qualitative changes were found in all 11 subjects (100%) with cerebellar diseases and in nearly all of their recordings; these changes, however, are test findings and not part of the list of objectives of this study. Our scope was not to statistically quantify and analyze these morphological changes; they may, however, become the object of a future study.

## DISCUSSION

We compared the qualitative parameters of horizontal saccades (stationary and moving) between normal subjects and patients with cerebellar conditions. The parameters that were investigated were velocity, latency and accuracy. Additionally, we added the variables sex and age to check the variations of the parameters above as a function of these other two variables in a control group and a study group (with cerebellar conditions).

The quantitative parameters of saccades in both groups (control and study) were unaltered; thus, according to our results, one cannot state that a patient has a cerebellar disease simply by analyzing velocity, latency and accuracy. Aschoff et al. also reported similar results in 1971^5^ showing that saccade velocity in monkeys was unaffected by injury of the cerebellar cortex.

On the other hand, saccades were qualitatively affected in all patients with cerebellar disorders. Findings included saccadic dysmetria (hypometria and hypermetria), correction saccades and glissades at a higher frequency in the study group compared to controls. This finding suggests that the cerebellum modulates saccade amplitude and operates compensatory mechanisms for saccades, because of the number of correction saccades in study group subjects. Optican and Robinson6 carried out full cerebellectomies in monkeys which resulted in saccade dysmetria without velocity and latency abnormalities.

Some authors define saccade dysmetria as altered saccade accuracy, while other suggest it is a change in saccade amplitude (Ganança et al^12^).

Correction saccades always accompany true saccade dysmetria. In hypermetria, corrective saccades are opposite to the displacement of the target, while in hypometria the initial saccade is small and correction saccades continue towards the target.

**Figure 2:**
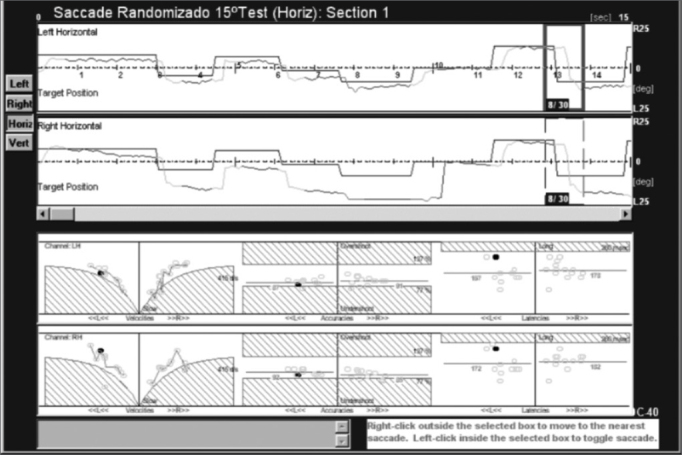
Saccades of a patient with cerebellar disease. See correction saccade hypometria.

Glissades are saccades that end gradually, rather than abruptly. They may be present in patients with myasthenia gravis, cerebellar injury (these patients are unable to adjust their pulse rates) and in patients with internuclear ophthalmoplegia^13^ (Fig. 2).

Patients with familial and degenerative diseases of the cerebellum also present saccade dysmetria, albeit with no specific pattern; the types of dysmetria vary among these patients, and may include correction saccades.

Zee et al. (1976) found abnormal saccades in two patients with spinocerebellar degeneration.^14^

**Figure 3:**
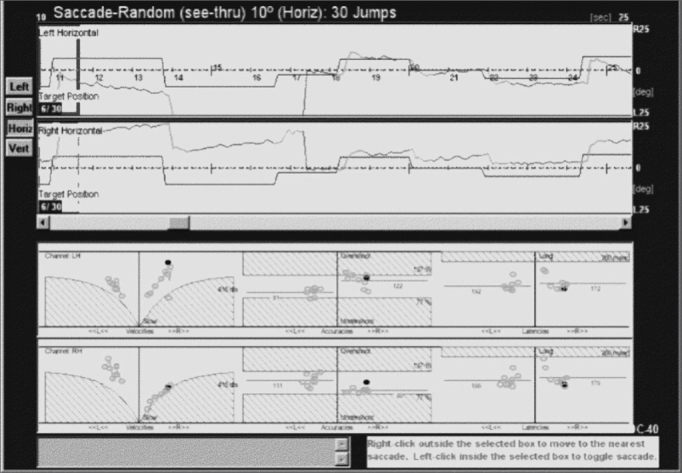
Saccades of a subject with familial cerebellar syndrome familiar.

Tracings of a patient with familial cerebellar syndrome who was included in this study are shown next (Fig. 3).

The patient was a young male adult presenting ataxic gait and unbalance. His father had the same symptoms and was also included in this study. The figure shows that this patient had hypermetria, hypometria, and correction saccades. Left and right eye tracings were significantly asymmetric. Quantitative parameters were within the normal range (see lower portion of the velocity, accuracy and latency charts). The frequency of saccade dysmetria (hypometria and hypermetria), correction saccades and glissades was higher in the study group compared to controls. This finding suggests that the cerebellum modulates saccade amplitude and helps compensate for saccades because of the number of correction saccades in study group subjects.

Several authors have considered saccade dysmetria as a clinical sign of cerebellar disease.

The presence of saccade dysmetria encountered in several studies of patients with cerebellar disease suggest that the cerebellum modulates the amplitude of each saccade and operates in adaptive and compensatory mechanisms for saccades, since subjects with cerebellar dysfunction had correction saccades following overshoot (saccades move beyond the target) or undershoot (saccades stop before the target position) of light targets in otoneurological tests.

The cerebellum does not affect peak velocity, latency or accuracy. For instance, the brainstem controls saccade peak velocity, while saccade latency depends on intact neural pathways in the brainstem, superior colliculus and other structures.

Zee and Leigh have suggested that the cerebellum appears to control saccade accuracy. Our findings do not support this statement, since there were no differences in accuracy among study and control group subjects, with accuracy values within normal limits.^4^

Neural pathways connecting the cerebellum and the saccade system have not been fully elucidated; this theme goes beyond the scope of this study. Several hypotheses have been raised to explain these pathways in an attempt to describe how the cerebellum affects saccades.

This study also showed that there were no differences in the three quantitative parameters (horizontal stationary and moving saccades) between males and females, both between study and control groups and between sexes within the same group.

These data show that sex does not affect saccade velocity, latency and accuracy, and that therefore the same normal range for these parameters may be applied to both sexes.

Dispersion chart analysis was used to study the variable age, again revealing no significant differences in the three parameters, both between study and control groups and between sexes within the same group.

Thus, the variable age does not affect saccade velocity, latency and accuracy measurements in both healthy subjects and patients with cerebellar conditions.

Higher latency values could be expected in older patients (≥ 60 years) according to North-American reference values in the Micromedial manual. This could not be confirmed in this study because the maximum age in the study group was 53 years and the maximum age in the control group was 50 years.

It may be inferred that the cerebellum connects with the saccade system, and that ENG - by studying the morphology of saccade recordings (stationary and moving) - can support otoneurologists in the early diagnosis of cerebellar dysfunction even when no changes appear in imaging methods; most of the patients in the study group had not radiologic findings in cerebellar morphology.

## CONCLUSION

Quantitative parameters (peak velocity, latency and accuracy) of horizontal saccades (stationary and moving) in patients with cerebellar conditions were similar to those of normal subjects, and are thus not helpful for assessing cerebellar syndromes.

Sex and age do not affect saccade quantitative parameters both within and among the control and study groups.
